# Histoplasmosis mimicking a tumor of the thumb in a patient with neurofibromatosis type I: A case report and review of the literature

**DOI:** 10.1002/ccr3.8088

**Published:** 2023-10-23

**Authors:** Joshua Marc Levine, Juan Manivel, Jeffery Luna

**Affiliations:** ^1^ University of Minnesota Medical School Minneapolis Minnesota USA; ^2^ VA Medical Center Minneapolis Minnesota USA

**Keywords:** hand, histoplasmosis, infection, neurofibromatosis, thumb

## Abstract

We describe an immunocompromised 73‐year‐old male with a history of neurofibromatosis type 1 (NF1) who presented with a lesion on the thumb concerning for malignancy that was found to be histoplasmosis. This unique case highlights the importance of a thorough history and a broad differential diagnosis in the management of new osteoarticular lesions.

## Graphical abstract

Posterior‐anterior view of right thumb showing extensive bony destruction and demineralization of first metacarpal head and proximal portion of proximal phalanx.

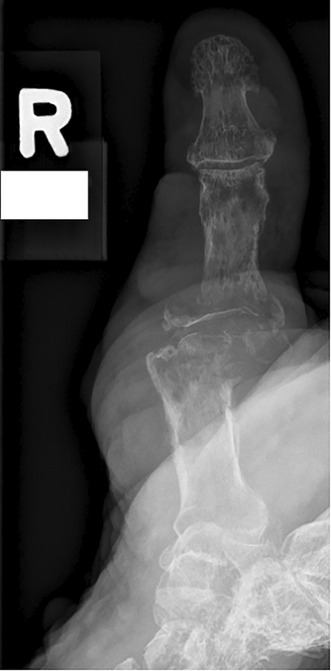

## 1 INTRODUCTION

Neurofibromatosis 1 (NF1) is a genetic disorder due to a mutation in the NF1 tumor suppressor gene, affecting 0.03%–0.04% of the general population. Patients with NF1 commonly present with cutaneous café‐au‐lait spots and neurofibromas. Malignant transformation of neurofibromas should be suspected when patients report substantial pain, rapid growth, or change in consistency of a neurofibroma. The risk of malignant transformation to neurofibrosarcomas (malignant schwannomas) has been reported at 8–13%.^1^ In immunocompromised patients, bacterial and fungal infections should be ruled out with the aid of laboratory studies, advance imaging, and tissue sampling.

*Histoplasma capsulatum var. capsulatum* (*H. capsulatum*) is a dimorphic fungus most commonly found in North and South America. It represents the most common cause of endemic fungal infection in the United States, with the highest rates of infection seen in the Ohio and Mississippi River Valleys; however, the true incidence of histoplasmosis is unknown as many patients are asymptomatic.^2^, ^3^ The fungus is commonly found in soil rich with bird and bat excrements and is highly associated with cave spelunking.^3^ *H. capsulatum* should not be confused with a closely related fungus, *Histoplasma capsulatum var. duboisii*, which causes African histoplasmosis and is almost exclusively found in Africa.^3^

Disseminated histoplasmosis is rare, representing about 0.05% of all cases.^4^ Infection by *H. capsulatum* often occurs via inhalation of microconidia, which are ingested by alveolar macrophages. The microconidia are converted to the yeast form, which replicate in macrophages. The infection can then spread to the lymph nodes and reticuloendothelial system.^5^ The majority of infections occur in immunocompetent asymptomatic individuals. However, large exposures to the conidia may lead to severe respiratory infection. When immunocompromised patients are exposed to *H. capsulatum*, severe infection and dissemination are more likely to occur.^3^

Clinical presentation of histoplasmosis is variable and includes asymptomatic, acute, chronic, and disseminated infection. Disseminated histoplasmosis may present as acute, subacute, and chronic forms. The acute form is associated with abrupt onset of systemic symptoms such as fever and occurs most commonly in immunocompromised patients. The subacute form manifests as focal lesions in several visceral organs. The chronic form has an indolent course with focal lesions in patients with effective cell‐mediated immune responses. Similar to *Mycobacterium tuberculosis*, *H. capsulatum* can reactivate; this has been well documented in patients taking immunosuppressive drugs.^3^ Reactivation of *H. capsulatum* manifesting as an osteoarticular infection has not been well described in the literature.

When histoplasmosis infection manifests in the musculoskeletal system, tendon involvement is more common than osteoarticular infection or osteomyelitis.^6^ In a systematic review of osteoarticular infections by dimorphic fungi from 1970 to 2012, 222 cases were identified. Of those cases, 8% (18 of 222) were caused by *H. capsulatum*. Thus, cases of disseminated osteoarticular histoplasmosis are rarely reported. To our knowledge, there have been no reports of osteoarticular *H. capsulatum* infection limited to the metacarpal phalangeal (MCP) joint.

The diagnosis of *H. capsulatum* is confirmed by histopathologic examination, microbiologic culture, or genetic tests using appropriate probes. On histopathology, necrotizing granulomas may resemble mycobacterial infection. Thus, appropriate stains are essential for diagnosis. Microbiologic cultures can take up to 6–8 weeks.^5^, ^7^

We present a unique case of a patient with NF1 and a history of disseminated histoplasmosis 20 years prior, who presented with swelling of the first metacarpophalangeal (MCP) joint and was found to have reactivated osteoarticular histoplasmosis. The concern for malignant transformation of a neurofibroma led to a prompt biopsy, diagnosis, and treatment of the infection. This case highlights the importance of obtaining a detailed infectious disease history and maintaining a broad differential diagnosis in the setting of new osteoarticular lesions.

## 2 CASE PRESENTATION

A 73‐year‐old male, with a history of NF1, seizure disorder, gastrointestinal stromal tumor, monoclonal gammopathy of undetermined significance, and Addison's disease from prior histoplasmosis infection requiring hydrocortisone replacement, presented to the orthopedic clinic for worsening right thumb pain and swelling that started about 2 months prior. The patient reported no trauma to the right hand but had noted some oozing from an area on his dorsal thumb MCP joint.

On examination, a 3.0 cm × 2.0 cm soft tissue mass with a 1 cm circular area of scab was noted on the dorsal aspect of the thumb. Café‐au‐lait spots along with multiple neurofibromatosis nodules were noted throughout the body and right hand. The patient was able to freely move the thumb at the carpometacarpal (CMC) joint but not at the distal interphalangeal (DIP) joint. His vital signs were within normal limits.

**FIGURE 1:**
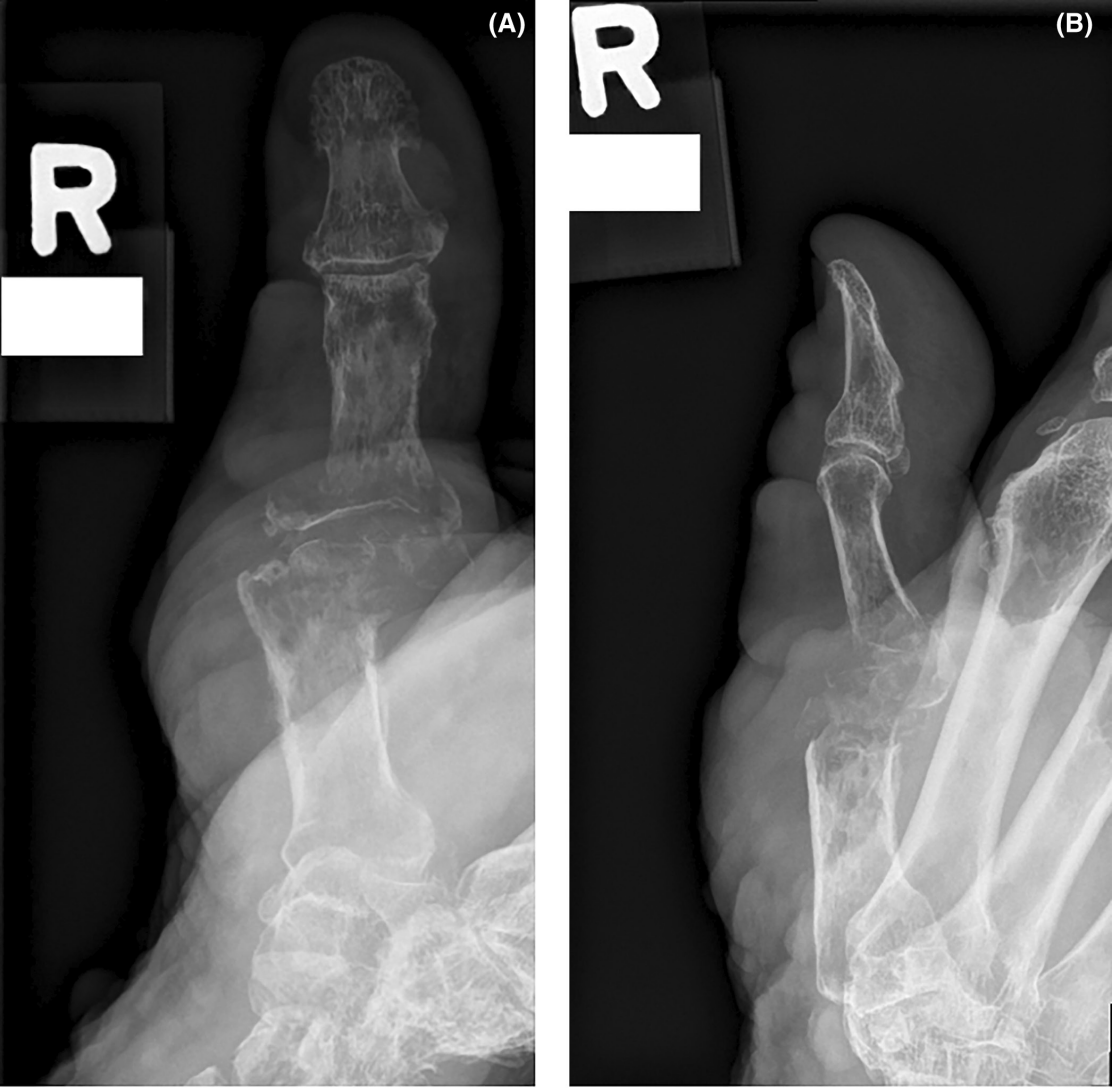
Radiographs right thumb. (A) Posterior–anterior view of right thumb (left image). (B) Lateral view of right thumb (right image).

Radiographs of the right thumb revealed a comminuted fracture with extensive bony destruction and demineralization of the first metacarpal head and proximal portion of the proximal phalanx (Figure 1A,B). Compared to previous right thumb radiographs taken 16 days earlier, significant progression of the pathological process was noted.

**FIGURE 2:**
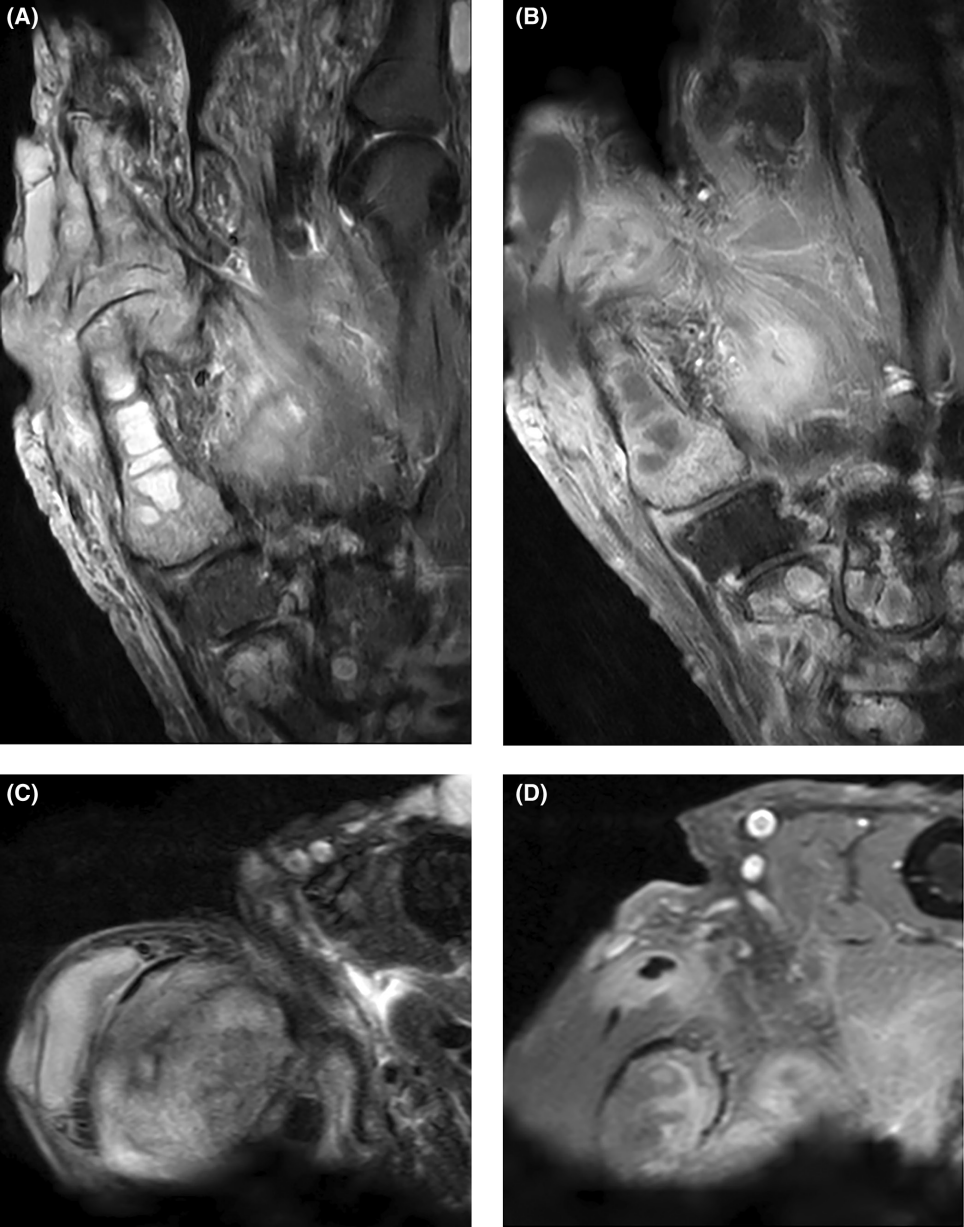
MRI right thumb *. (A) Coronal STIR without contrast (top left image). (B) Coronal T1 with contrast (top right image). (C) Axial T2 without contrast (bottom left image). (D) Axial T1 with contrast (bottom right image). (E) *note there is significant motion artifact.

The differential diagnoses included right thumb neurofibrosarcoma and septic arthritis. An MRI (Figure 2A–D) revealed abnormal bone marrow signal with enhancement within the proximal phalanx and first metacarpal of the thumb. Irregular enhancement with central nonenhancement suggested fluid collection in the first webspace, as well as possible subcutaneous fluid collection dorsal to the proximal phalanx of the thumb. Mild tenosynovitis of the deep flexor tendons was also noted. The radiologic differential diagnosis of infiltrative neoplasm versus infection led to a decision to proceed with tissue biopsy for definitive diagnosis.

The operative procedure was initiated utilizing a dorsal approach over the first MCP joint, creating a 0.5 × 0.5 cm open wound with a 15 blade. Dissection was carried down to the subcutaneous layer. Scant necrotic tissue with predominantly pale firm tissue was found, along with MCP joint erosion. The sinus tract was excised. A wedge biopsy was obtained of flesh‐colored nodular tissue over the MCP joint. The specimens were sent for cultures and frozen sections. After obtaining the specimens, 2 g of cefazolin were given. Hemostasis was achieved. Frozen sections showed inflammation and no evidence of malignancy. The wound was then closed with nylon sutures and a sterile dressing was applied.

**FIGURE 3:**
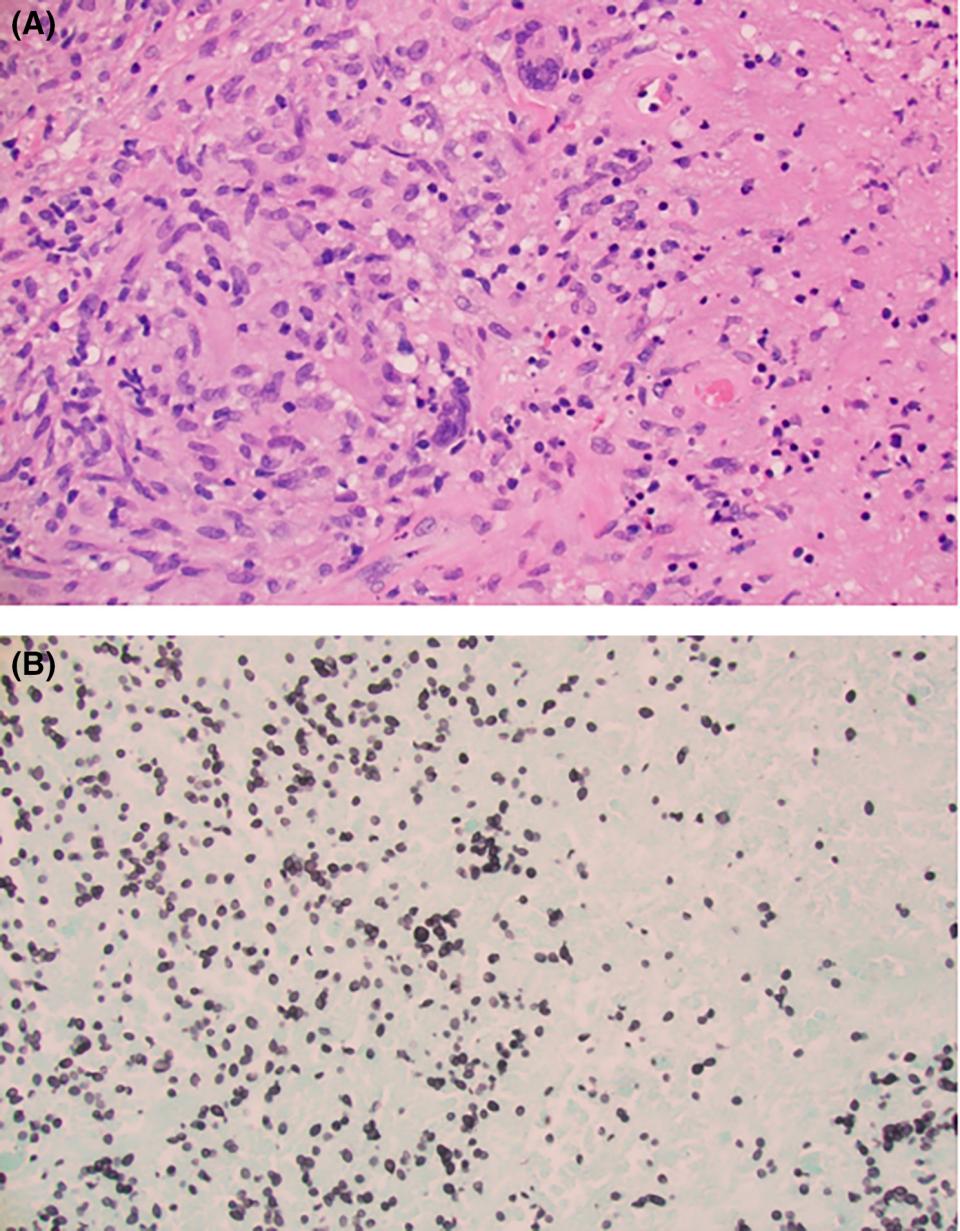
Biopsy histology. (A) Hematoxylin and eosin stain, magnification 400X (left image). (B) Gomori methenamine silver stain, magnification 600X (right image).

Final histopathology revealed necrotizing granulomas (Figure 3 **)**. In Figure 3A, necrotizing granuloma consists of necrosis (right) surrounded by palisading epithelioid histiocytes and multinucleated giant cells. In Figure 3B, innumerable histoplasma yeast organisms are identified. The findings of necrotizing granulomatous inflammation with numerous yeast organisms are those of histoplasmosis. Gram stain of direct smear showed neutrophils and budding yeasts. Microbiologic culture in Sabouraud dextrose media was suggestive of *H. capsulatum*. Isolate referred to the Minnesota Department of Health using a Histoplasma probe was positive for *H. capsulatum*. Acid fast stain, mycobacterial and anaerobe cultures revealed no organisms.

The patient returned to clinic 2 weeks after surgery and was doing well. He was informed that cultures grew *H. capsulatum*. Treatment options discussed included a two‐stage resection arthroplasty and antifungal treatment. At that time, the COVID‐19 pandemic restricted all elective surgeries, so tentative surgical plans were postponed.

Three weeks later, the patient presented to the infectious disease clinic; he reported no pain in the right thumb and an open ulcer was noted. A detailed review of the patient's medical history revealed disseminated histoplasmosis about 20 years prior. The infection involved his lungs and adrenal glands. He did not recall specific antifungal agents received but had been on hydrocortisone replacement for Addison's disease since. The infectious disease physician suggested the histoplasmosis likely represented reactivation of the infection, given his remote history. CT scan of the chest, abdomen, and pelvis from the time of initial presentation to the orthopedic clinic was reviewed. Two small calcified granulomas in the left apex were noted with scarring and atelectasis in the lateral and basal segments of the right lower lobe and lateral segment of the right middle lobe. No acute infiltrates or pleural effusions were identified. No signs of active lung infection were observed.

The patient was prescribed itraconazole solution 200 mg three times daily for 3 days, then twice daily. The duration of itraconazole treatment would likely be lifelong given reactivation of the disease. The physician ordered liver function tests, along with urine Histoplasma antigen and itraconazole trough levels to be taken 4 weeks later. Additionally, as antifungals alone are often insufficient for treatment, the infectious disease consultant recommended that joint arthroplasty be performed at a later time, unless indicated earlier for pain.

Six weeks later, the patient returned to the infectious disease clinic with clinical improvement other than diarrhea that was attributed to itraconazole. He was switched from itraconazole liquid to capsules (200 mg twice daily), which relieved the diarrhea. At 9 weeks postoperative, he contacted the orthopedic clinic with complaints of increased draining and shortening of his right thumb, hoping both could be addressed through surgery. He continued to be pain‐free. A preoperative visit was scheduled; however, he canceled the appointment.

Approximately 9 months after first presenting to the orthopedic clinic, the patient presented to the emergency department with shortness of breath and was found to be COVID‐19 positive with concomitant lactic acidosis. He was admitted to the medical intensive care unit. Itraconazole 200 mg twice daily was continued. He developed hypoxic respiratory failure and was placed on BiPAP with prone positioning. As his condition deteriorated, the goals of his care focused on comfort; he succumbed to COVID‐19 complications 2 weeks after admission. An autopsy was not performed.

## 3 DISCUSSION

In our patient with known NF1, there was high clinical suspicion for malignant transformation. The MRI findings were inconclusive and supported the need for biopsy. In the operating room, gross examination revealed a solid lesion without purulence. Malignant transformation could not be ruled out until after histopathology confirmed an infectious etiology.

MRI is useful to identify neurofibrosarcoma size and location. Recently, F‐fluorodeoxyglucose (F‐FDG)‐PET scans have been used as a highly sensitive and specific method for detecting malignant transformation. However, diagnosis must be confirmed with biopsy. Surgery is the only curative treatment for neurofibrosarcomas.^8^

Few reports of *H. capsulatum* affecting the hand exist in the literature. In a systematic review of histoplasmosis in the upper extremity conducted between 1992 and 2015, only 15 cases were identified. Eight of those cases presented with tenosynovitis, five with osteomyelitis, one with an abscess, and one as carpal tunnel syndrome. Most cases involved the arm and wrist with only three cases involving a metacarpal, digit, or MCP joint. One patient, a 44‐year‐old HIV‐positive woman with immune reconstitution inflammatory syndrome, had involvement of the metacarpal bones, but the infection was bilateral and diffuse, affecting all 10 metacarpals. She was initially treated with IV amphotericin, followed by oral itraconazole, but ultimately refused treatment and died from septic shock.^9^ Another patient was a 48‐year‐old with a history of Sjogren's syndrome on prednisone, who had Histoplasma tenosynovitis of the thumb. This patient was effectively treated with open carpal tunnel release and tenosynovectomy, followed by IV amphotericin and itraconazole.^10^ Lastly, a 42‐year‐old female with SLE presented with tenosynovitis of the index finger with swelling of the PIP and MCP joints, as well as swelling between the second and third MCP joints. She was effectively treated with oral itraconazole for 12 months with complete resolution of the symptoms.^11^ None of the patients in these cases presented with isolated infection of the MCP joint.

Treatment of osteoarticular *H. capsulatum* infection requires antifungal agents, typically amphotericin B followed by triazole drugs.^7^ Triazole antifungal agents inhibit the synthesis of ergosterol, a principal fungal cell membrane sterol.^7^ Amphotericin B works by binding to ergosterol, causing membrane disruption, leakage of cell contents, and cell death. Combination therapy of triazole and amphotericin B has been reported as an effective treatment for osteoarticular fungal infections; however, there may also be a role in the use of echinocandins, an antifungal drug class that works by inhibiting synthesis of beta‐1,3‐D‐glucan, another key molecule in the fungal cell wall.^7^ Surgical treatment may be necessary; in such cases, the lesion should be excised, followed by treatment with antifungal therapy. Bone reconstruction and arthroplasty can be utilized after the pathogen has been cleared to restore musculoskeletal function.^7^

This report presents an unusual case of reactivated osteoarticular *H. capsulatum* infection. In our case, the patient had a remote history of pulmonary histoplasmosis that disseminated to the adrenal glands approximately 20 years prior. Primary lung lesions have rarely been reported to disseminate to the bone and joints. *H. capsulatum* osteomyelitis progresses slowly, from 7 days to 3 years, with a median time to diagnosis of 90 days.^7^ Chronic steroid use in our patient may have contributed to *H. capsulatum* reactivation. To our knowledge, no cases of *H. capsulatum* infection isolated to the MCP joint have been reported.

A history of NF1 in our patient led to prompt indication for tissue biopsy. In other cases of *H. capsulatum* involving the upper extremity and lower concerns for malignancy,^12^ time to diagnosis of infection can be prolonged, often after months of intraarticular steroid injections. If a patient with a history of dimorphic fungal infection such as histoplasmosis, presents with a new osteoarticular lesion, fungal infection should be considered early, especially if the patient is immunosuppressed.

Additionally, this case is unique because isolated *H. capsulatum* osteoarticular infection mimicked possible malignant transformation of a neurofibroma. This suspicion should remain high in patients with NF1 and often necessitates tissue biopsy. To our knowledge, there have been no reported cases of *H. capsulatum* infection that presented clinically as a tumor in the hand.

A significant limitation of this study is the lack of follow up due to the patient's demise from COVID‐19 infection. Documentation of the patient's recovery on the antifungal regimen would have added to the discussion of the treatment of reactivated osteoarticular *H. capsulatum* infection. Additionally, reconstruction with two‐stage resection arthroplasty was considered. If the patient had survived and proceeded with operative care, documentation of the success and challenges of the surgery would have brought additional insight to the use of arthroplasty to treat a *H. capsulatum* osteoarticular infection of the hand.

## 4 CONCLUSION

This report describes reactivated osteoarticular *H. capsulatum* infection isolated to the MCP joint. The case is unique because the clinical presentation and history of NF1 raised a high suspicion for malignant transformation of a neurofibroma, which was found to be a lesion caused by reactivated *H. capsulatum*. Additionally, to our knowledge, no cases of *H. capsulatum* infection isolated to this particular anatomic location have been documented. This case report highlights the importance of a thorough history of previous infections and maintaining a broad differential in the management of new osteoarticular lesions. Further research is warranted to investigate the effectiveness of surgical and nonsurgical treatments for osteoarticular histoplasmosis, particularly of the hand. Additionally, further research may provide insight into the epidemiology of this rare manifestation of histoplasmosis infection.

## AUTHOR CONTRIBUTIONS

**Joshua Marc Levine:** Data curation; investigation; methodology; writing – original draft; writing – review and editing. **Juan Manivel:** Conceptualization; resources; writing – review and editing. **Jeffery Luna:** Conceptualization; project administration; supervision; writing – review and editing.

## FUNDING INFORMATION

There was no financial support or other contributions for this work.

## CONFLICT OF INTEREST STATEMENT

The authors have no conflict of interest to declare.

## CONSENT

Written informed consent was obtained from the patient's next of kin to publish this report in accordance with the journal's patient consent policy.

## ETHICS STATEMENT

Ethical approval was not required for the case report as per the country's guidelines.

## Data Availability

The data that support the findings of this study are available from the corresponding author upon reasonable request.
